# Supplementary material for: Charge polarity-dependent ion-insertion asymmetry during electrochemical doping of an ambipolar π-conjugated polymer
Source: Nat Commun. 2022 Dec 16;13:7788. doi: 10.1038/s41467-022-35408-w (PMC9758163; doi:10.1038/s41467-022-35408-w)
Supplement: Supplementary file 1 — Supplementary Information [file 41467_2022_35408_MOESM1_ESM.pdf]

Supplementary Information

Charge Polarity-Dependent Ion-Insertion Asymmetry During Electrochemical Doping of an Ambipolar π-Conjugated Polymer

Jibin J. Samuel, Ashutosh Garudapalli, Chandrasekhar Gangadharappa, Smruti Rekha Mahapatra, Satish Patil, Naga Phani B. Aetukuri*

Solid State and Structural Chemistry Unit, Indian Institute of Science, Bengaluru – 560012, Karnataka, India

*Corresponding Author: Naga Phani B. Aetukuri: phani@iisc.ac.in

Table of Contents

1. *Determination of HOMO/LUMO levels of DPP-based polymers* 2

2. *Transfer characteristics of 2DPP-OD-TEG OECTs as a function of anion* 4

3. *Cyclic voltammetry experiments – discussion on parasitic side-reactions* 6

4. *Electrochemical Impedance Spectroscopy of 2DPP-OD-TEG thin films* 10

5. *Cation-dependent transfer characteristics of 2DPP-OD-TEG OECTs* 12

6. *Electrochemical Impedance Spectroscopy of 2DPP-OD-TEG thin films* 14

7. *Dependence of electrochemical doping on hydrophilicity and hardness of ions* 15

8. *Electrochemical doping of 2DPP-OD-TEG with large ions* 16

9. *Evolution of absorbance spectra for n- and p-type electrochemical doping* 19

10. *Relation between ion transfer free energy and Gutmann DN/AN* 20

11. *Ion size dependent transfer characteristics of 2DPP-OD-HEX polymer* 22

12. *Passive swelling of 2DPP-OD-TEG in aqueous electrolyte* 24

13. *Correlation between n-type OECT operation and glycol sidechain weight fraction* .. 25

14. *Donor and acceptor numbers of selected solvent molecules organized according to functional group* 25

1. Determination of HOMO/LUMO levels of DPP-based polymers

HOMO/LUMO electronic levels were determined using CV experiments performed in a nitrogen glove box at a voltage sweep rate of 100 mV/s. Polymer films electrodes, obtained by spin coating a 10mg/mL solution of 2DPP-OD-TEG or 2DPP-OD-HEX in 3:2 v/v mixture of 1,1,2,2-tetrachloroethane and chloroform on ITO/glass substrates, were used as working electrodes. A 0.1 M tetrabutylammonium hexafluorophosphate (TBA-PF_6) dissolved in acetonitrile (ACN) was used as the electrolyte. WE potential was referenced to a custom-built Ag^+/Ag reference electrode with 0.1 M TBA-PF_6/0.01Ag-OTf in ACN as fill solution. The reference electrode (RE) potential was calibrated using a ferrocenium/ferrocene (Fc^+/Fc) redox couple (0.089 V vs. the Ag^+/Ag RE). A platinum wire was used as the counter electrode (CE).

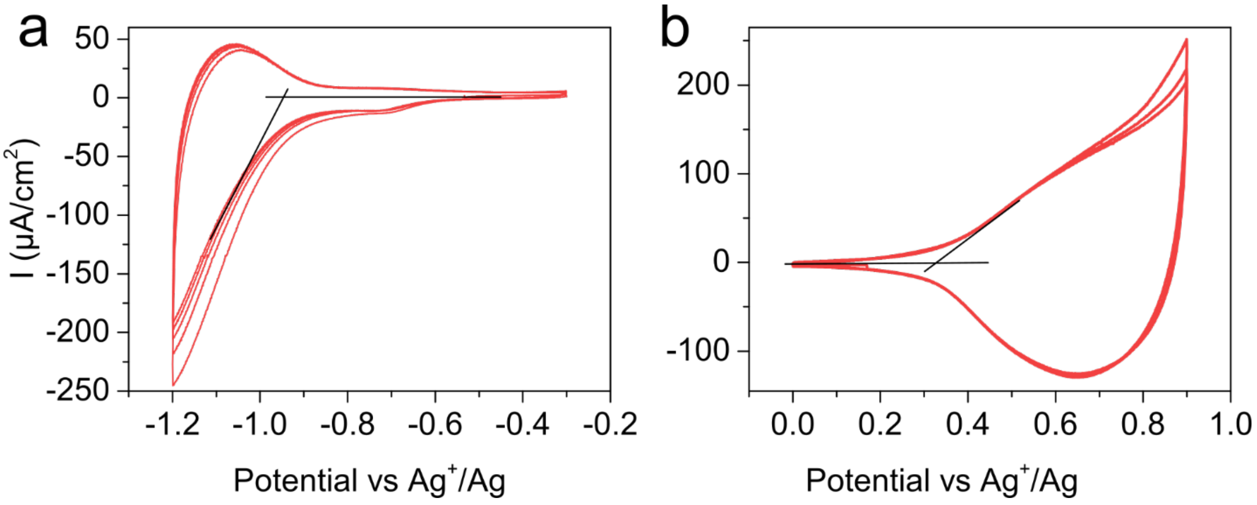

Supplementary Figure 1. Cyclic voltammograms (CVs) of 2DPP-OD-TEG films performed over (a) a voltage range of -1.2 V to -0.3 V and (b) over a voltage of 0 V to 0.9 V vs. Ag^+/Ag at a scan rate of 100 mV/s.

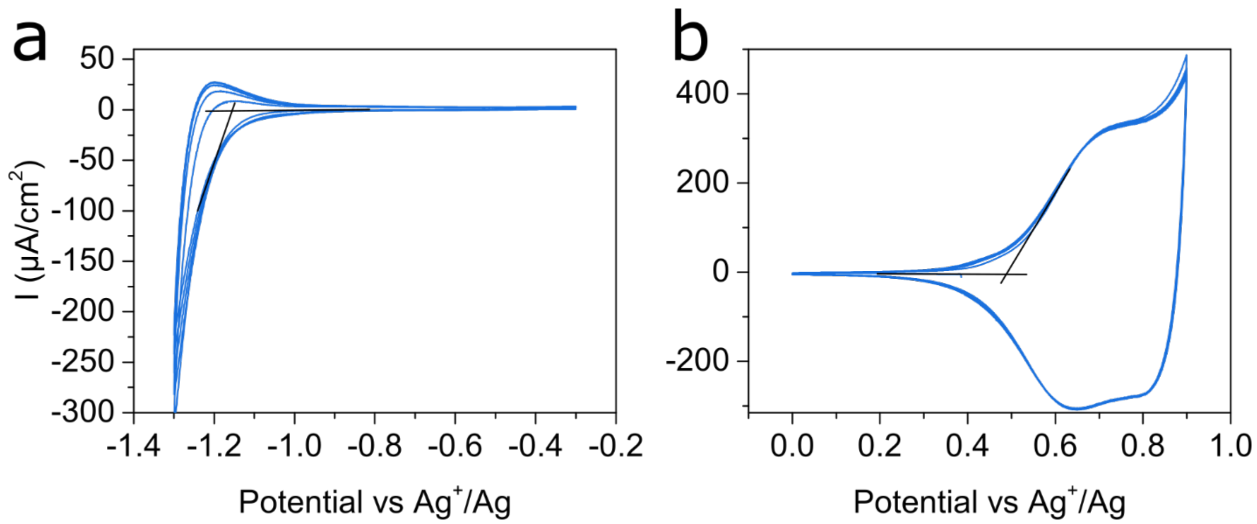

Supplementary Figure 2. CVs of 2DPP-OD-HEX films performed over (a) a voltage range of -1.3 V to -0.3 V and (b) over a voltage of 0 V to 0.9 V vs. Ag^+/Ag at a scan rate of 100 mV/s.

The onset potentials of the reduction and oxidation waves ($E_{Red,onset}$, $E_{Ox,onset}$) were used to determine the HOMO and LUMO positions of the polymers using the formulae given by equations S1 and S2

$$E_{HOMO} = -\left[E_{Ag/Ag^+}(Ox,onset) - E_{Ag^+/Ag}(Fc^+/Fc) + E_{SHE}(Fc^+/Fc) + 4.44\right] \quad (S1)$$

$$E_{LUMO} = -\left[E_{Ag/Ag^+}(red,onset) - E_{Ag^+/Ag}(Fc^+/Fc) + E_{SHE}(Fc^+/Fc) + 4.44\right] \quad (S2)$$

The Ag^+/Ag reference electrode potential was calibrated in a 0.1 M $TBAPF_6$ in acetonitrile containing a small amount of ferrocene. The $E_{1/2}$ for ferrocenium/ferrocene redox, $E_{Ag+/Ag}$ (Fc^+/Fc) was determined to be 0.16 V versus Ag^+/Ag reference electrode potential. E_{SHE} (Fc^+/Fc) was assumed to be at 0.65 V on the standard hydrogen electrode (SHE) potential scale.[1]

Polymer	Red. onset (V vs Ag/Ag⁺)	Ox. onset (V vs Ag/Ag⁺)	E_{LUMO} (eV)	E_{HOMO} (eV)
TEG	-0.95	0.32	-3.98	-5.25
HEX	-1.15	0.49	-3.78	-5.42

Supplementary Table 1. 2DPP-OD-TEG and 2DPP-OD-HEX reduction and oxidation onsets and calculated HOMO and LUMO energies from CV experiments in 0.1 M $TBAPF_6$ in acetonitrile

2. Transfer characteristics of 2DPP-OD-TEG OECTs as a function of anion

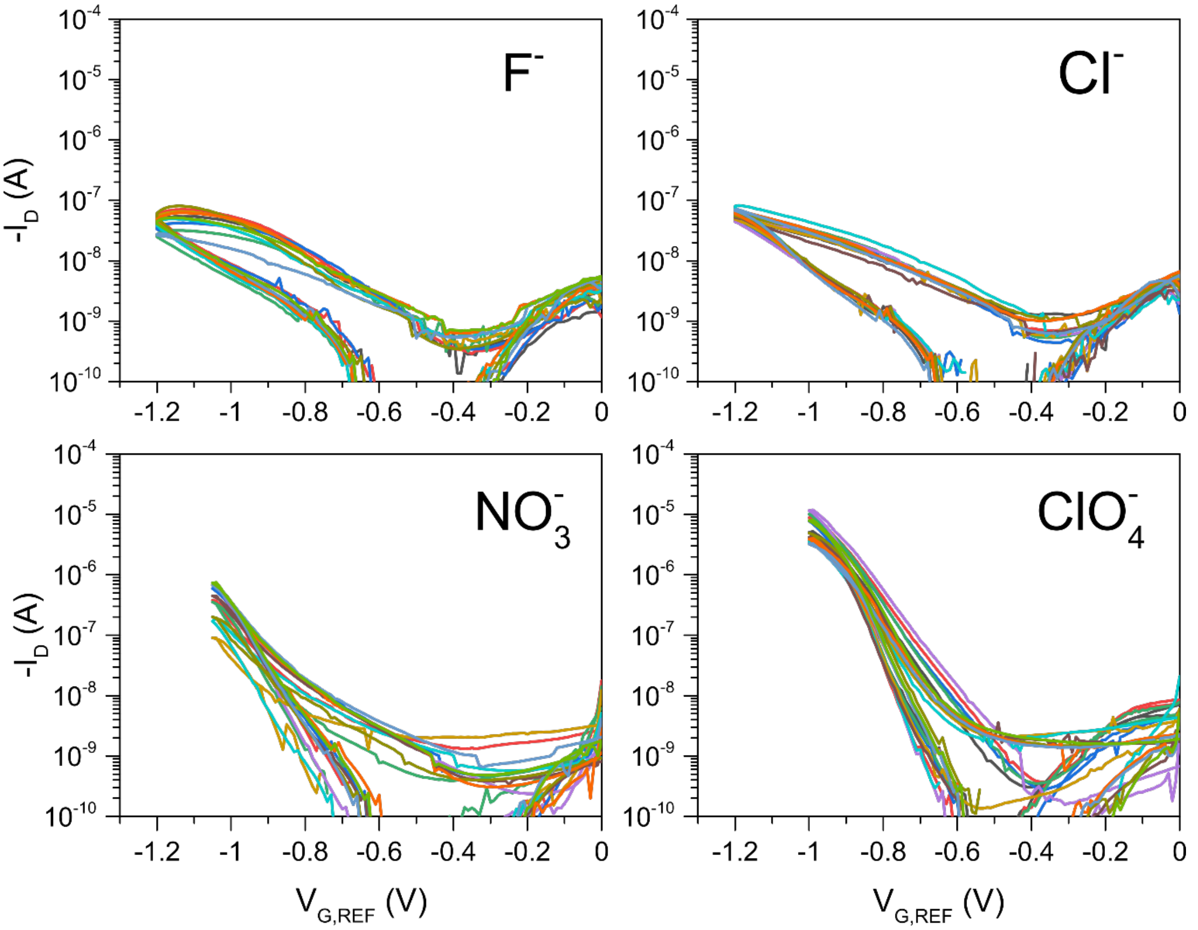

Supplementary Figure 3. Transfer characteristics of individual OECTs measured in 0.1 M aqueous electrolytes of sodium salts with 4 different anions. At least 10 different OECT devices were measured in each of the electrolytes. The individual measurements are represented by different colors of the line graphs.

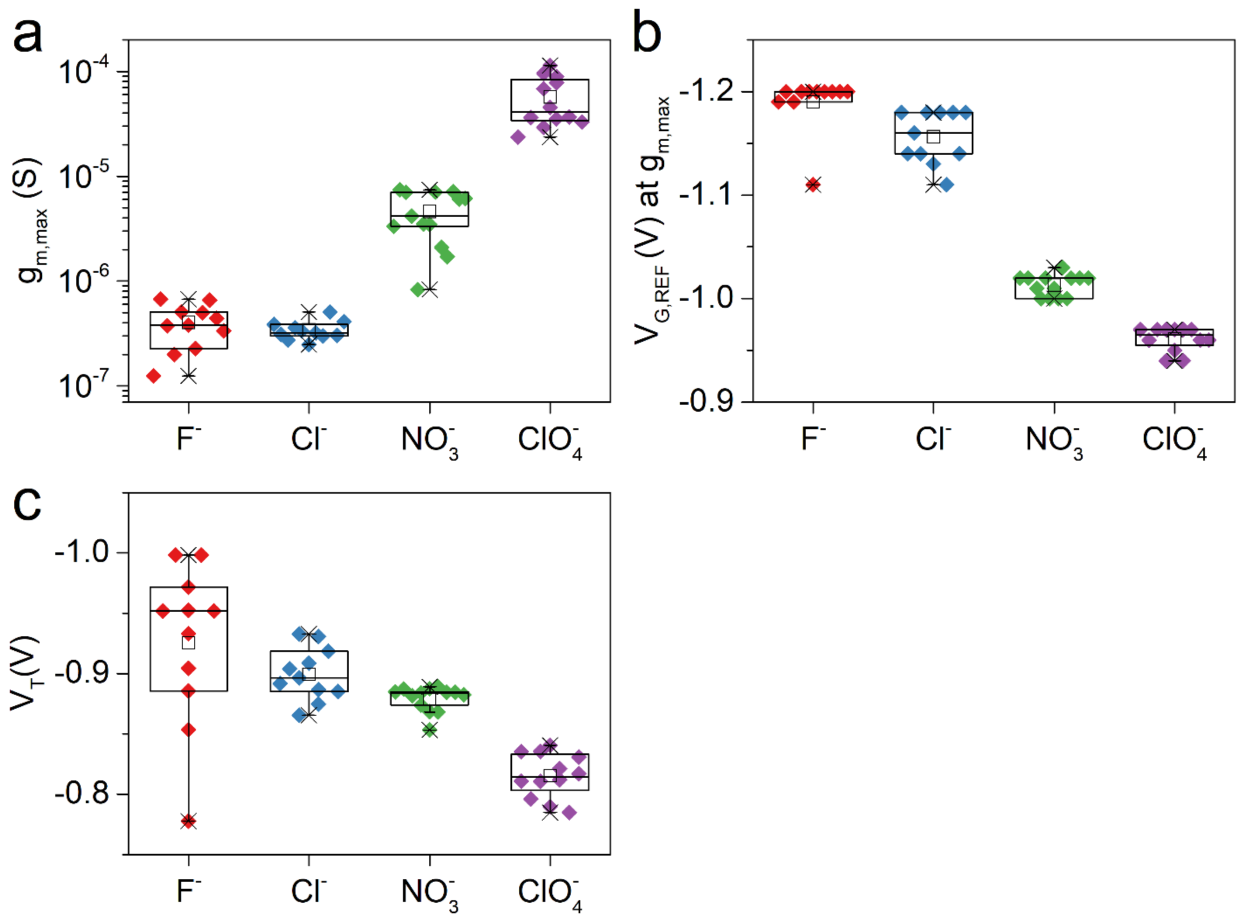

Supplementary Figure 4. Box plots showing the distribution of measured p-OECT device parameters, each point representing an individual device. Box plots of (a) maximum transconductance, (b) the $V_{G,REF}$ value at which the maximum value of transconductance occurs, and (c) p-type threshold voltage extracted from the intercept of linear portion of $I_D^{0.5}$ vs. $V_{G,REF}$ with the x-axis for different anions. For the box plots, center line is median; box limits are 25th and 75th percentiles; whiskers are outliers within 25th and 75th percentile + 1.5x interquartile range; '□' represents mean value; '×' represents maximum and minimum values.

3. Cyclic voltammetry experiments – discussion on parasitic side-reactions

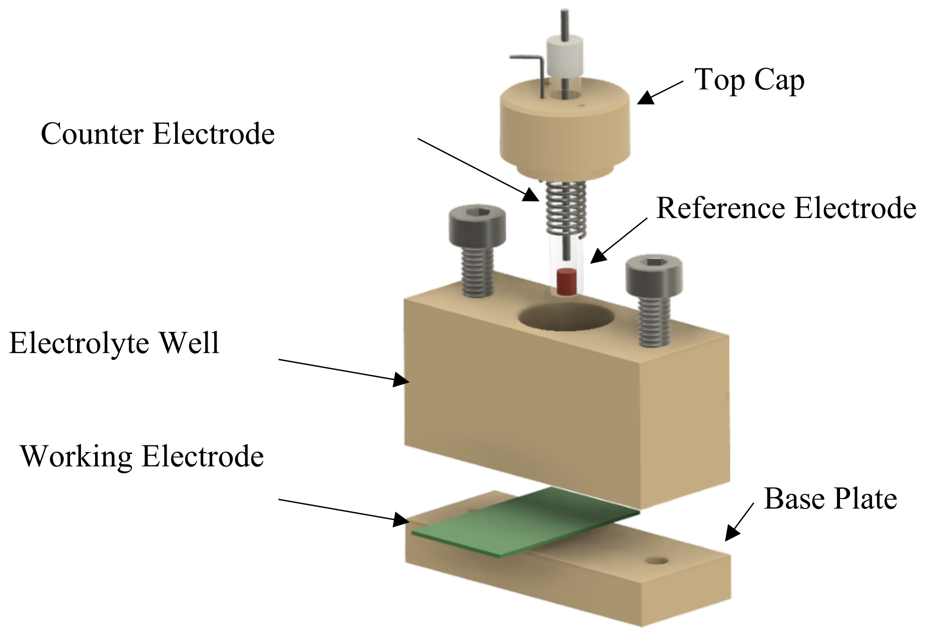

Supplementary Figure 5. 3-electrode cell for CV/EIS measurements

"The potentials used for OECT and CV experiments for p-type electrochemical doping lie outside the electrochemical stability window of water which correspond to the range of -0.649 V to +0.580 V vs. Ag/AgCl (1M NaCl fill solution) at pH = 7. At oxidative potentials of the order of 1 to 1.2 V vs. Ag/AgCl used for the p-type electrochemical doping experiments, the oxygen reduction reactions (ORR)[2] given by equations 1 and 2 (redox potentials referenced to Ag/AgCl with 1M NaCl fill solution) occur in the backward direction resulting in O_2 evolution:

$$O_2 + 2H^+ + 2e^- \rightleftharpoons H_2O_2, E^0_{O_2/H_2O_2} = 0.033\,V \quad (1)$$

$$O_2 + 4H^+ + 4e^- \rightleftharpoons 2H_2O, E^0_{O_2/H_2O} = 0.580\,V \quad (2)$$

The redox reaction involved in p-type doping is given by equation 3.

$$P^+ + e^- \rightleftharpoons P^0, E^0_{P^+/P} = 0.58V \quad (3)$$

Since the reaction, $2P + O_2 + 2H^+ + 2e^- \rightleftharpoons 2P^+ + H_2O_2$ has E_{redox} = 0.033 – 0.58 = – 0.55 V < 0 , it is not feasible thermodynamically. In other words, the deep HOMO level of 2DPP-OD-TEG at -5.25 eV implies H_2O_2 generation by ORR in ambient conditions is suppressed (see Supplementary Fig. 6).[3] As a result, the reaction involving O_2 evolution from H_2O_2 can be discounted. The relevant parasitic reaction during p-type electrochemical doping of 2DPP-OD-

TEG is therefore decomposition of H_2O evolving O_2 and generating H^+ which might result in acidification of the electrolyte. However, the kinetic overpotentials for water oxidation on polymeric surfaces are known to be high.[2] Therefore, the fraction of charge that leads to the acidification of the electrolyte is expected to be small.

The parasitic water decomposition/oxygen evolution reaction can cause de-doping of the channel through the reaction given by equation 4 (also see Supplementary Fig. 6).

$$4\{P^+ : B^-_{Poly}\} + 2H_2O \rightleftharpoons 4P + 4B^-_{sol} + O_2 + 4H^+ \qquad (4)$$

To ensure that this is not the reason for lack of p-type doping with smaller anions such as F^- and Cl^-, we performed CV experiments in different anion containing electrolytes to quantify the parasitic reactions. CVs were measured on 2DPP-OD-TEG films at 100 mV/s scan rate from 0.2 V to an upper vertex potential corresponding to the maximum OECT gate voltages (see Supplementary Fig. 7 a-d).

An upper bound on the absolute amount of electrochemically generated parasitic side-products per cycle (ΔQ_{Irrev}) is calculated from the difference between the integrated charge under the oxidative (Q_{Ox}) and reductive (Q_{Red}) waves in the CV curve ($\Delta Q_{Irrev} = Q_{Ox} - Q_{Red}$). The measured ΔQ_{Irrev} is <10 µC even at an oxidation potential of 1.2 V in NaCl and NaF electrolytes (see Supplementary Fig. 7e). This corresponds to ~0.1 nano mole of the parasitic side-products generated per cycle (the volume of the electrolyte used in OECT experiments is ~ 1ml) . The corresponding change in pH due to the acidification of the electrolyte, if any, is insignificant around pH = 7. Further, ΔQ_{Irrev} is a small fraction of the total oxidative charge deposited during CV (see Supplementary Fig. 7f) and absolute value of ΔQ_{Irrev} for the larger anions is higher than for the smaller ions (see Supplementary Fig. 7e). Furthermore, the maximum oxidative charge (Q_{Ox}) for both F^- and Cl^- ion-insertion at 1.2 V are 25 µC and 14 µC, which is far below the Q_{Ox} of 95 µC and 42 µC respectively for ClO_4^- and NO_3^- at 1 V and 1.05 V. Notably, de-doping as a parasitic pathway is insufficient to explain the low charge insertion for electrochemical doping with F^- and Cl^-. This implies that the parasitic side-reactions do not play any significant role in the observed ion-size dependence during p-type doping of 2DPP-OD-TEG.

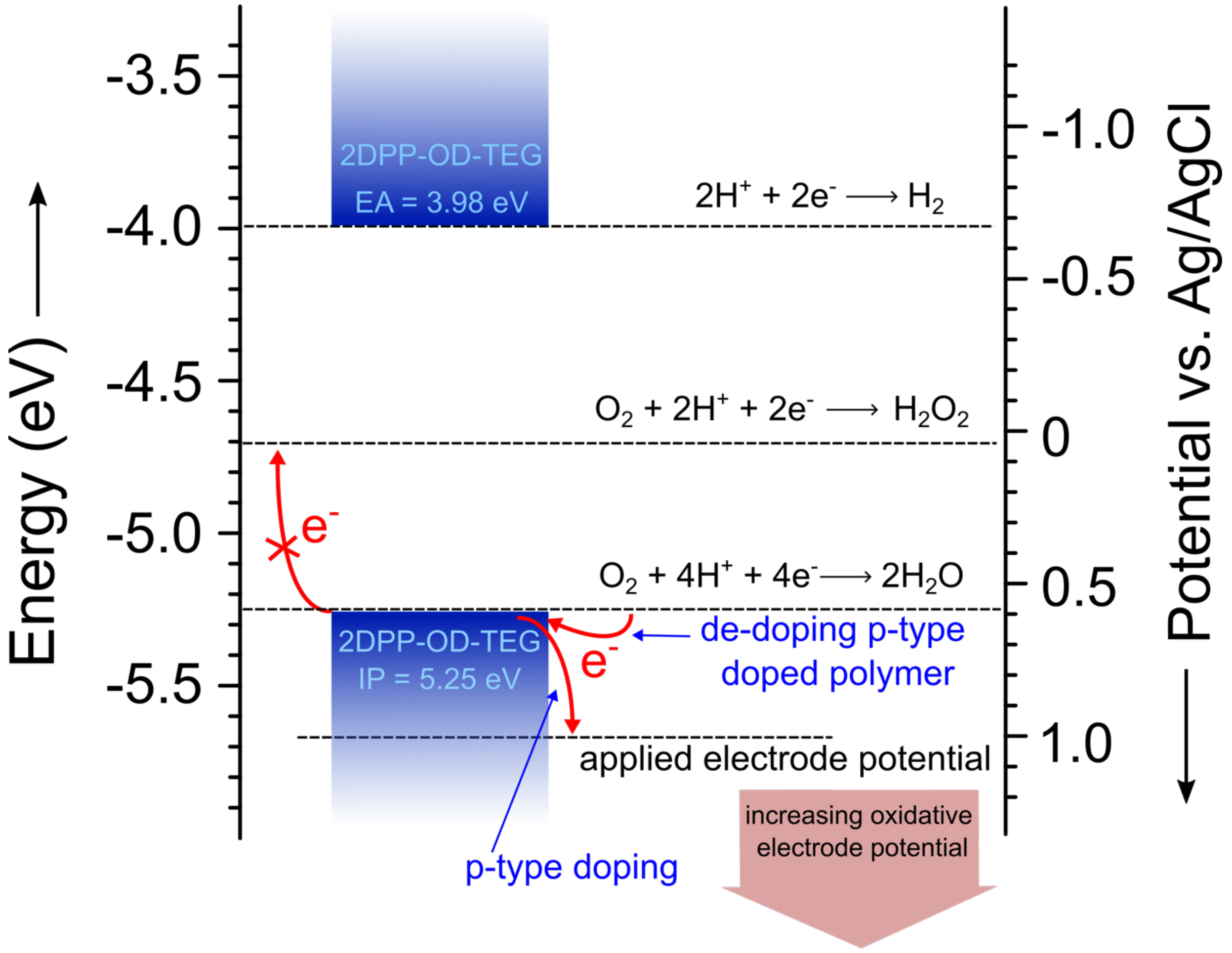

Supplementary Figure 6. Schematic showing the hydrogen evolution and oxygen evolution reactions (ORR) along with polymer Ionization Potential (IP) and Electron Affinity (EA) values. The neutral state of the polymer is stable against H_2O_2-generating ORR reaction because of the deep HOMO level of 2DPP-OD-TEG. However, the p-doped state can be dedoped by the parastic reactions involving oxidation of water to O_2.

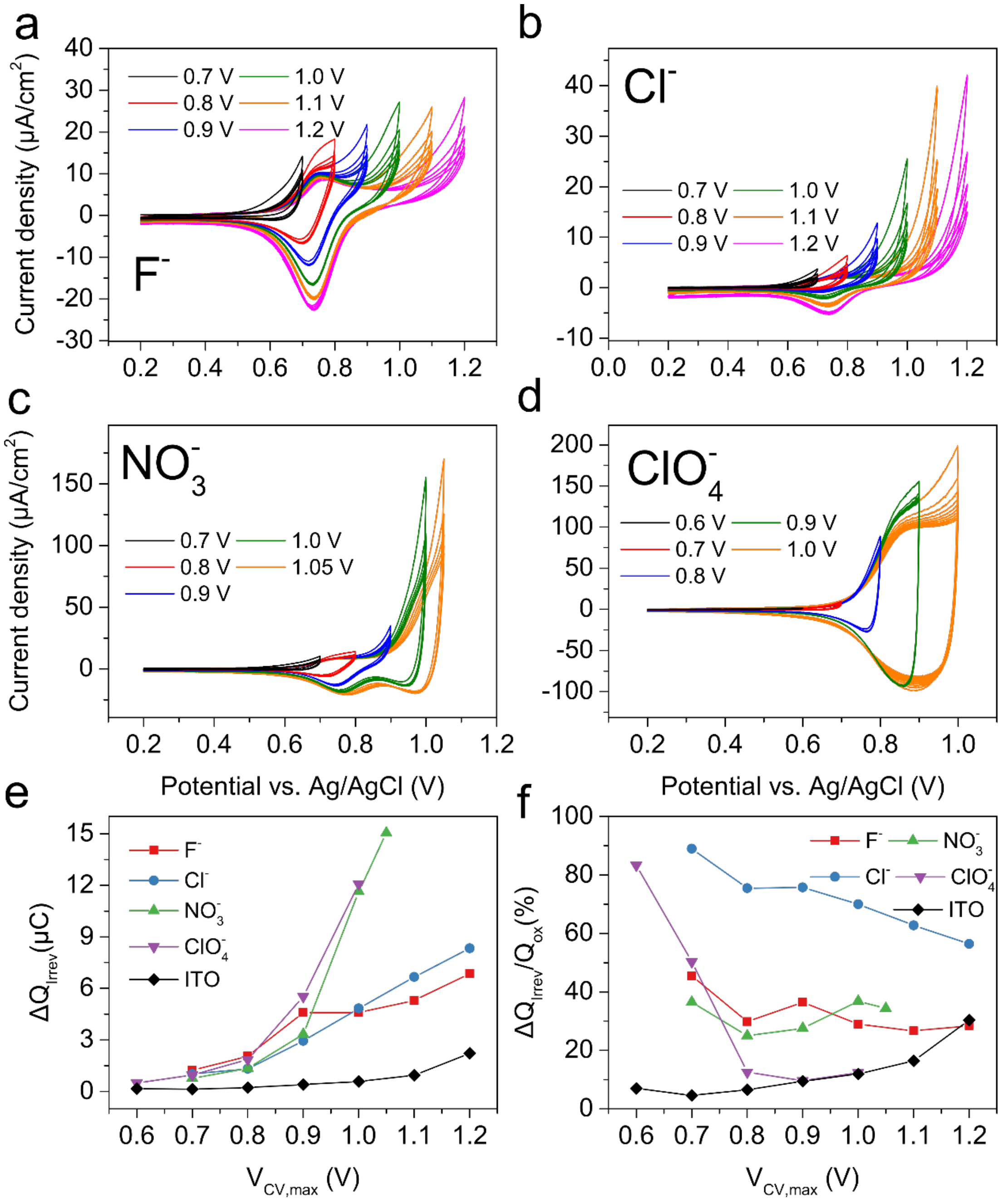

Supplementary Figure 7. (a-d) 5 cycles of cyclic voltammograms at 100 mV/s in electrolytes containing different anions going up to increasing maximum potentials, $V_{CV,max}$ (e) Mean irreversible charge, ΔQ_{irrev} (corresponding to parasitic electrochemical reactions) as a function of $V_{CV,max}$ calculated from the difference between the charge under oxidative and reductive waves. $\Delta Q_{irrev} \sim 7, 8, 15, 12, 2\ \mu C$ for F^-, Cl^-, NO_3^-, ClO_4^- and blank ITO (in NaCl) respectively at the highest used $V_{CV,max}$. (f) Percentage of ΔQ_{irrev} relative to Q_{ox}, the mean oxidative charge deposited during the oxidative wave of the CV, as a function of $V_{CV,max}$.

4. Electrochemical Impedance Spectroscopy of 2DPP-OD-TEG thin films

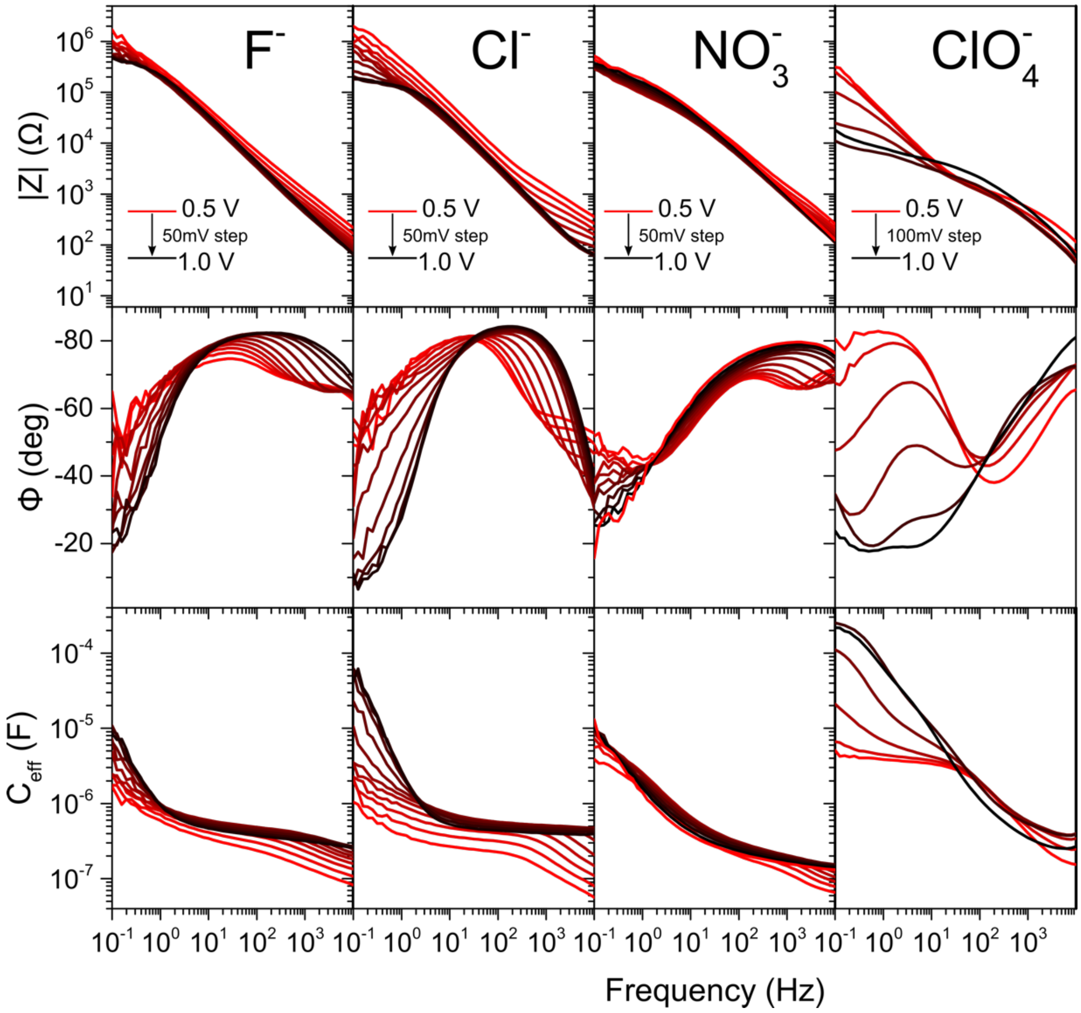

Supplementary Figure 8. Bode magnitude plots, Bode phase plots, and effective capacitance (C_{eff}) of 2DPP-OD-TEG thin films coated on ITO/Glass substrates as a function of applied voltage (a darker shade represents a more oxidative potential) measured in 0.1 M aqueous solutions of sodium salts with 4 different anions. C_{eff} shown in the bottom panel was calculated using the imaginary part of the impedance response using the equation $C_{eff} = -1/2\pi f Z_{img}$.

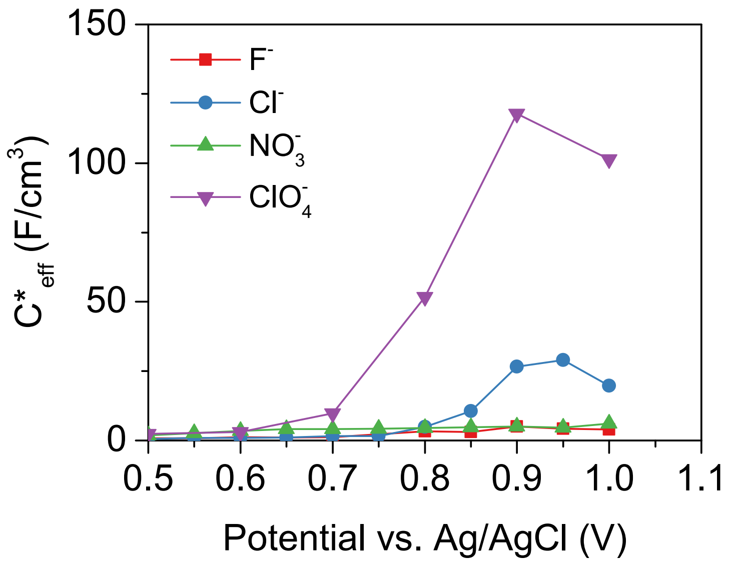

Supplementary Figure 9. Effective volumetric capacitance, C^*_{eff} by normalizing C_{eff} at 0.1Hz to film volume, as a function of potential for different anions

5. Cation-dependent transfer characteristics of 2DPP-OD-TEG OECTs

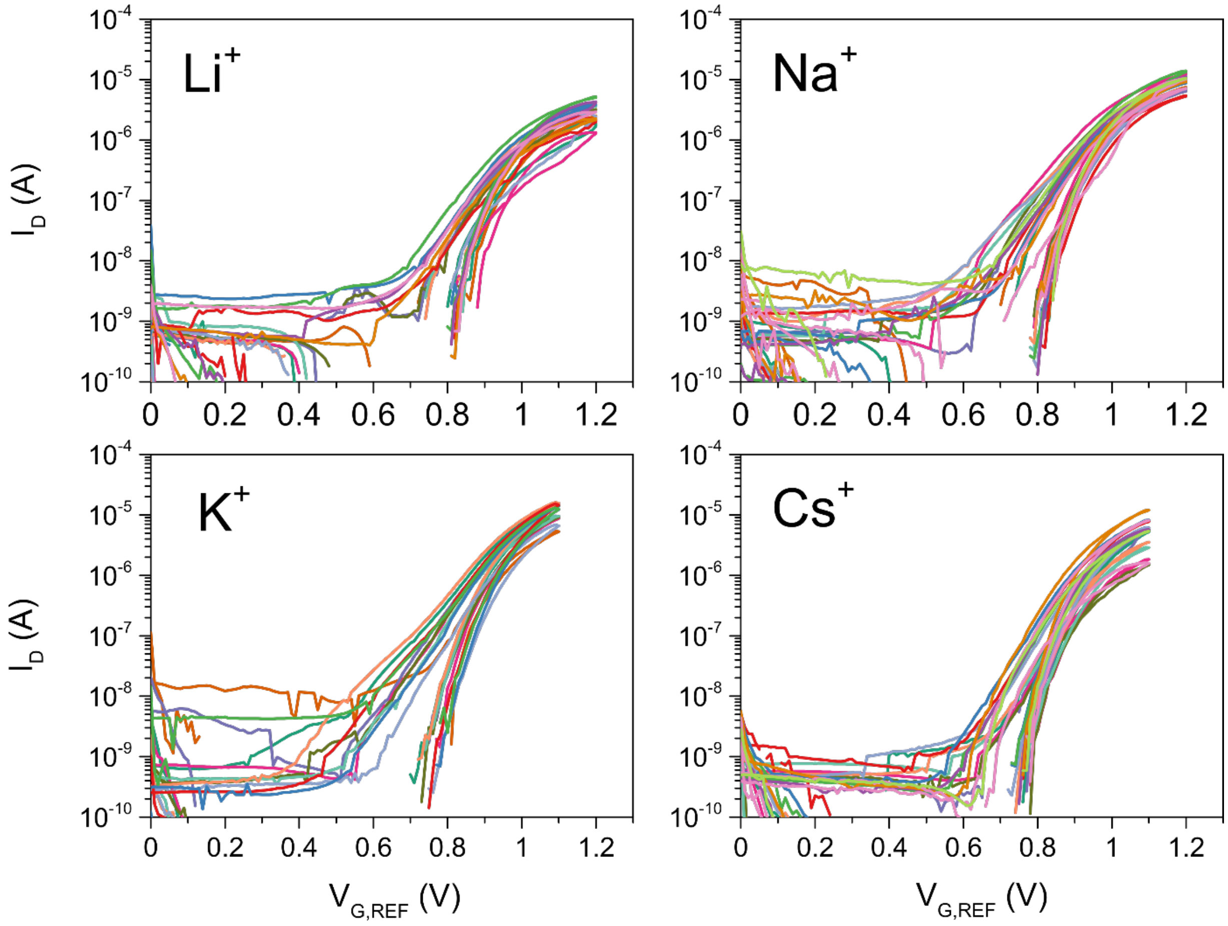

Supplementary Figure 10. Transfer characteristics of individual OECTs measured in 0.1 M aqueous electrolytes of chloride salts with 4 different cations. At least 10 different OECT devices were measured in each of the electrolytes. The individual measurements are represented by different colors of the line graphs.

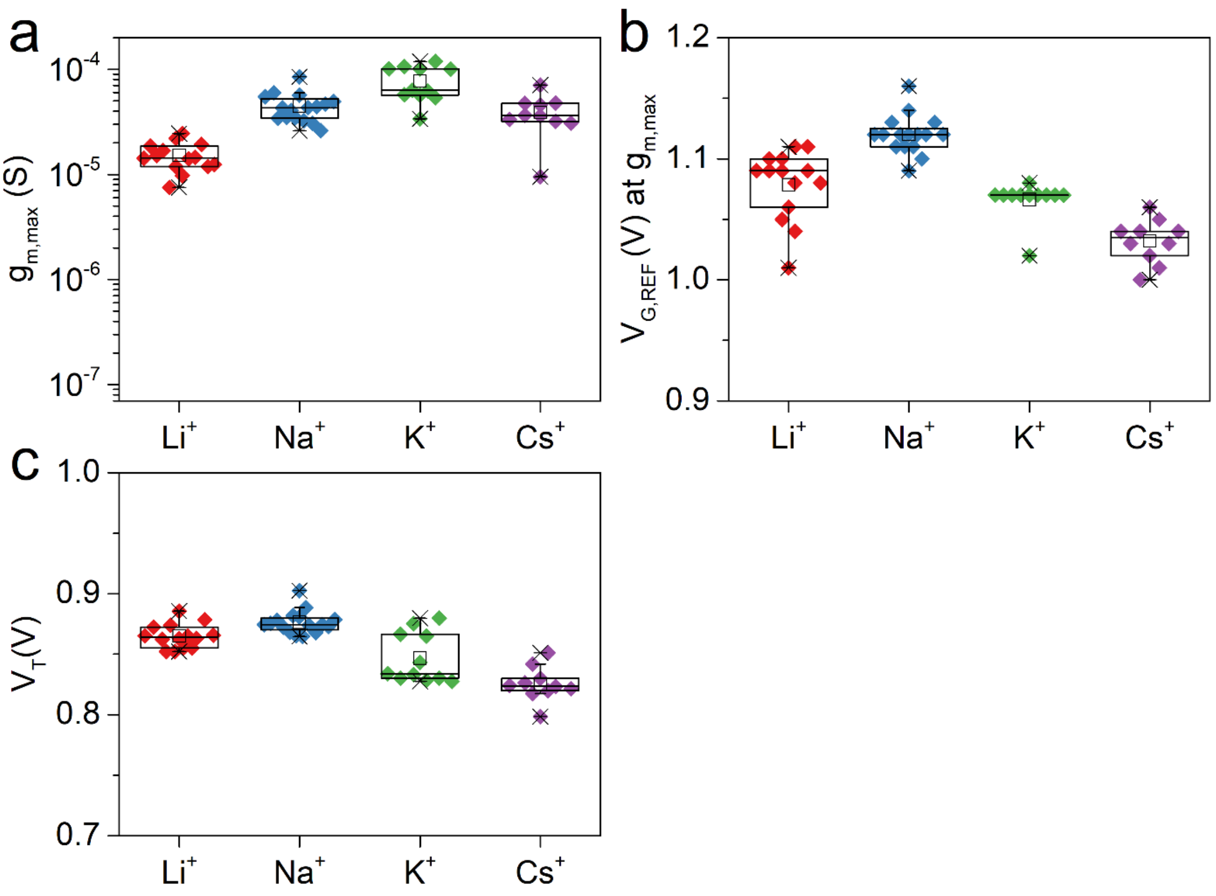

Supplementary Figure 11. Box plots showing the distribution of measured n-OECT device parameters, each point representing an individual device. Box plots of (a) maximum transconductance, (b) the $V_{G,REF}$ value at which the maximum value of transconductance occurs, and (c) n-type threshold voltage extracted from the intercept of linear portion of $I_D^{0.5}$ vs. $V_{G,REF}$ with the x-axis, for different cations. For the box plots, center line is median; box limits are 25th and 75th percentiles; whiskers are outliers within 25th and 75th percentile + 1.5x interquartile range; '□' represents mean value; '×' represents maximum and minimum values.

6. Electrochemical Impedance Spectroscopy of 2DPP-OD-TEG thin films

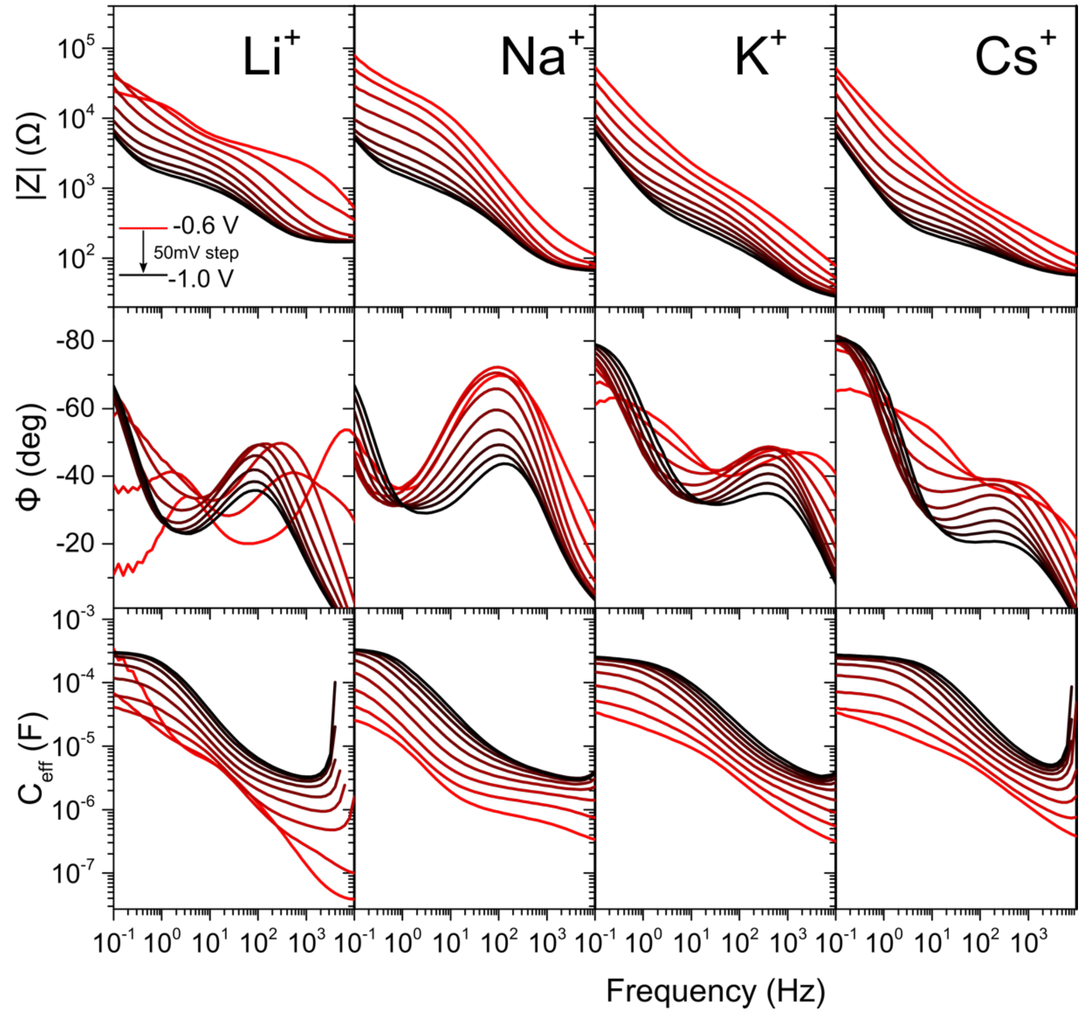

Supplementary Figure 12. Bode magnitude plots, Bode phase plots, and effective capacitance (C_{eff}) of 2DPP-OD-TEG thin films coated on ITO/Glass substrates measured at different applied voltages from 0.6 V to 1.0 V in increments of 50 mV (a darker shade represents a more reductive potential) in 0.1 M aqueous solutions of chloride salts with 4 different cations. C_{eff} shown in the bottom panel was calculated using the imaginary part of the impedance response using the equation: $C_{eff} = -1/2\pi f Z_{img}$.

7. Dependence of electrochemical doping on hydrophilicity and hardness of ions

The concept of hydrophilicity of an ion or the affinity of the ion for water can be quantified in terms of the free energy of hydration (ΔG_{hyd}) of the ion. ΔG_{hyd} is inversely correlated with the size of the ion.[4] The hardness of the ion is also correlated with ionic size – hardness decreases with increasing ionic size for both cations and anions. Hardness is quantified by the Pearson hardness, η.[5] Thus, effects of both hydrophilicity/hydrophobicity and hardness/softness, being correlated with ion size, are also indirectly accounted for in ion-size dependence. To further emphasize this, OECT $I_{D,max}$ is plotted in terms of ΔG_{hyd} and η of the dopant ions in Supplementary Fig. 13.

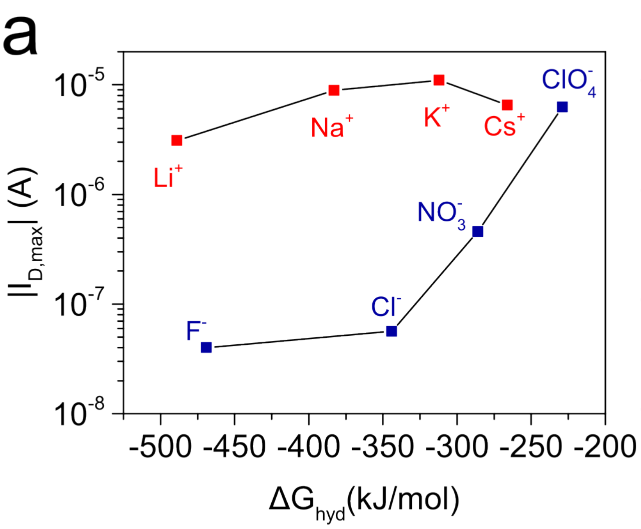

Supplementary Figure 13. Mean $I_{D,max}$ as a function of (a) free energy of hydration of ions[4] and (b) Pearson hardness[5]. Pearson hardness numbers for ClO_4^- and NO_3^- are not readily available.

8. Electrochemical doping of 2DPP-OD-TEG with large ions

Ion-insertion was measured for TBA^+ and OTF^- ions which are much larger than the cations and anions, respectively, considered for the studies presented in the main manuscript. For electrochemical doping with TBA^+, OECTs were gated through 0.1 M TBACl electrolyte solution and n-type operation was observed albeit with a lower maximum drain current of 462 ± 165 nA (at $V_{G,REF}$ = 0.9 V) compared to other cations (see Supplementary Figs. 14a, b). The devices were not stable at high gate voltages beyond 0.9 V. Further, the OECTs doped with TBA^+ showed a lower n-type threshold voltage of 0.69 ± 0.03 V compared to other cations. CVs of 2DPP-OD-TEG films in 0.1 M TBACl show an earlier onset of reduction compared to other cations which correlates with the lower n-type OECT threshold voltages (Supplementary Fig. 14c). Further, the CVs show a reductive peak current which corresponds to higher C^*_{eff} values obtained during EIS experiments suggesting a higher doping density with TBA^+ relative to other cations (Supplementary Fig. 14d).

Spectroelectrochemistry experiments performed during n-type doping with TBA^+ show a stronger quenching of the main absorption band and higher absorption in the polaron band (Supplementary Fig. 14e). Also, the absorption increases at relatively less negative reductive potentials compared to other cations which corroborates the observation of earlier reduction onset in CVs and higher doping densities in EIS.

In the case of p-type electrochemical doping with OTf^-, the OECTs show $I_{D,max}$ of ~ 4 μA which is slightly lower than that obtained during doping with ClO_4^- (Supplementary Figs. 15a,b). The peak currents observed with CV and C^*_{eff} values obtained using EIS are similar to that observed with ClO_4^- (Supplementary Fig. 15c,d). Absorbance changes with OTf^- show similar features but the absorbance changes are smaller (Supplementary Fig. 15e). Our observations suggest that the effectiveness of electrochemical doping decreases at very large dopant sizes potentially due to the larger distortion of the polymer matrix likely leading to disruption of inter-molecular interactions.

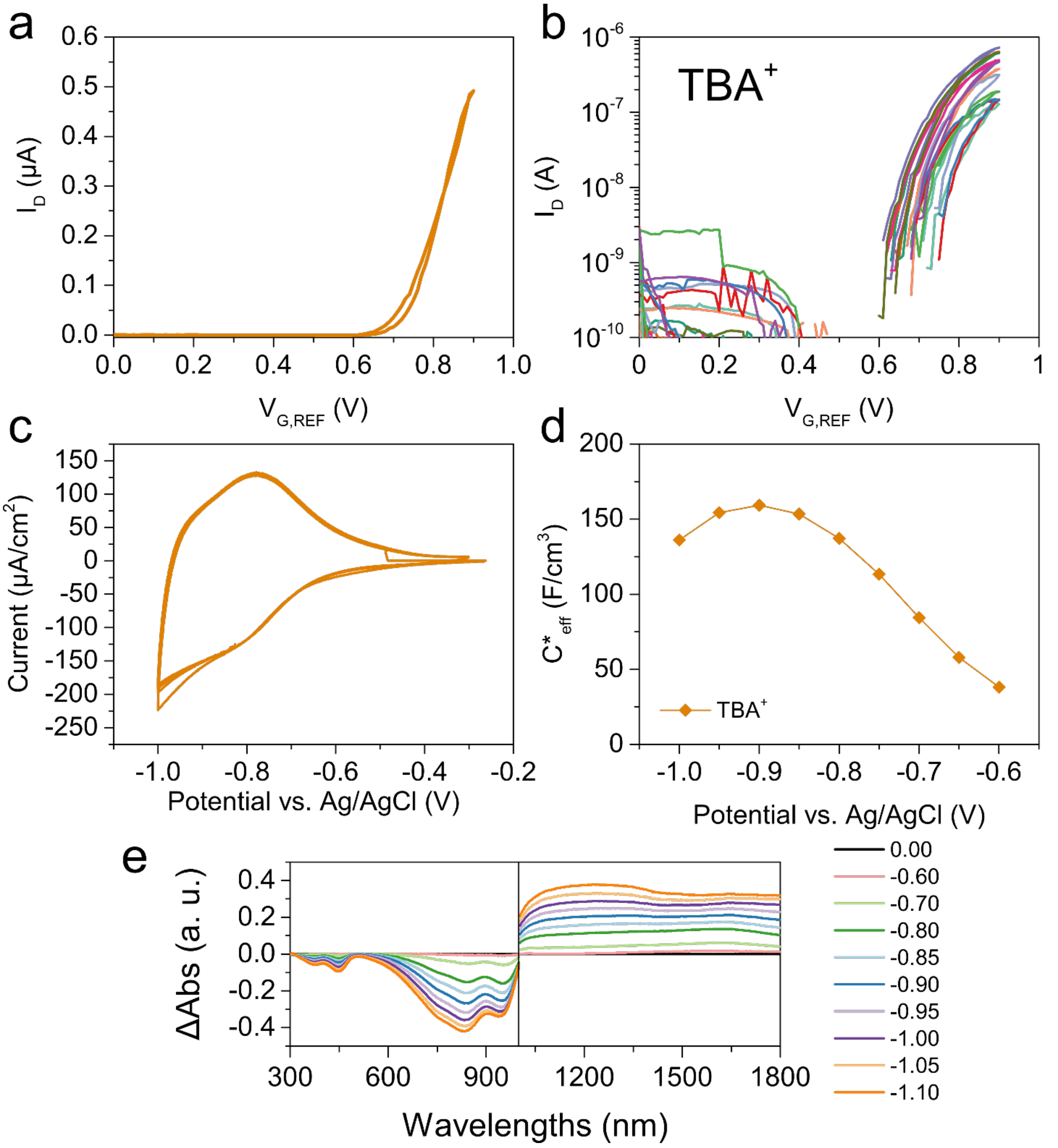

Supplementary Figure 14. OECT, CV, EIS and spectroelectrochemistry studies of 2DPP-OD-TEG during n-type electrochemical doping in 0.1 M aqueous TBACl solution (a) Typical n-OECT transfer characteristics (b) Log plots of n-OECT transfer characteristics measured for multiple OECTs. The different colors represent measurements done on different devices (c) 5 cycles of CV at 100 mV/s (d) Volumetric capacitance extracted from EIS measurements as a function of applied voltage (e) Evolution of relative absorption (relative to 0 V) as a function of applied voltage

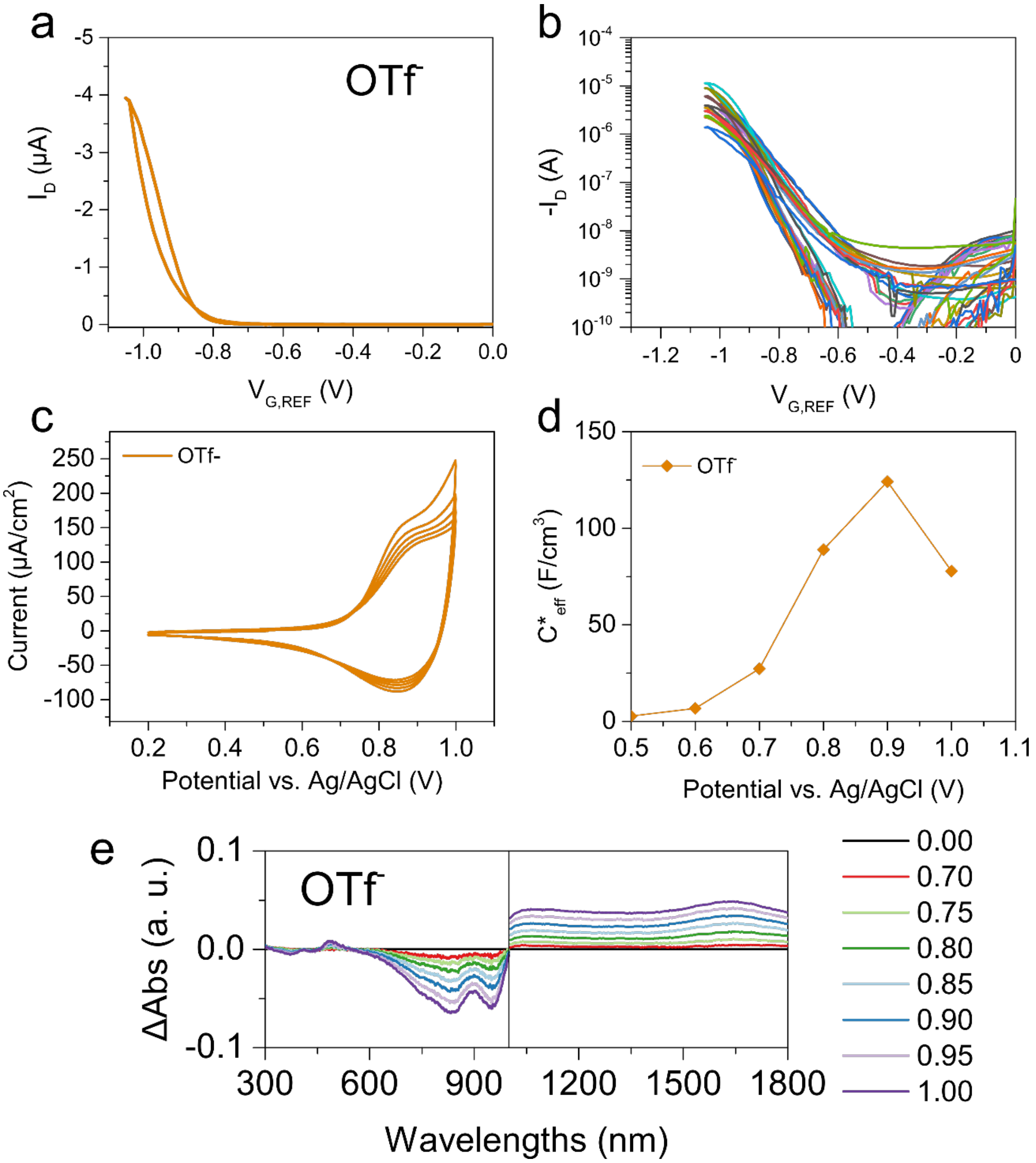

Supplementary Figure 15. OECT, CV, EIS and spectroelectrochemistry studies of 2DPP-OD-TEG during p-type electrochemical doping in 0.1 M aqueous NaOTf solution (a) Typical p-OECT transfer characteristics (b) Log plots of p-OECT transfer characteristics measured for multiple OECTs. The different colors represent measurements done on different devices (c) 5 cycles of CV at 100 mV/s (d) Volumetric capacitance extracted from EIS measurements as a function of applied voltage (e) Evolution of relative absorption (relative to 0 V) as a function of applied voltage.

9. Evolution of absorbance spectra for n- and p-type electrochemical doping

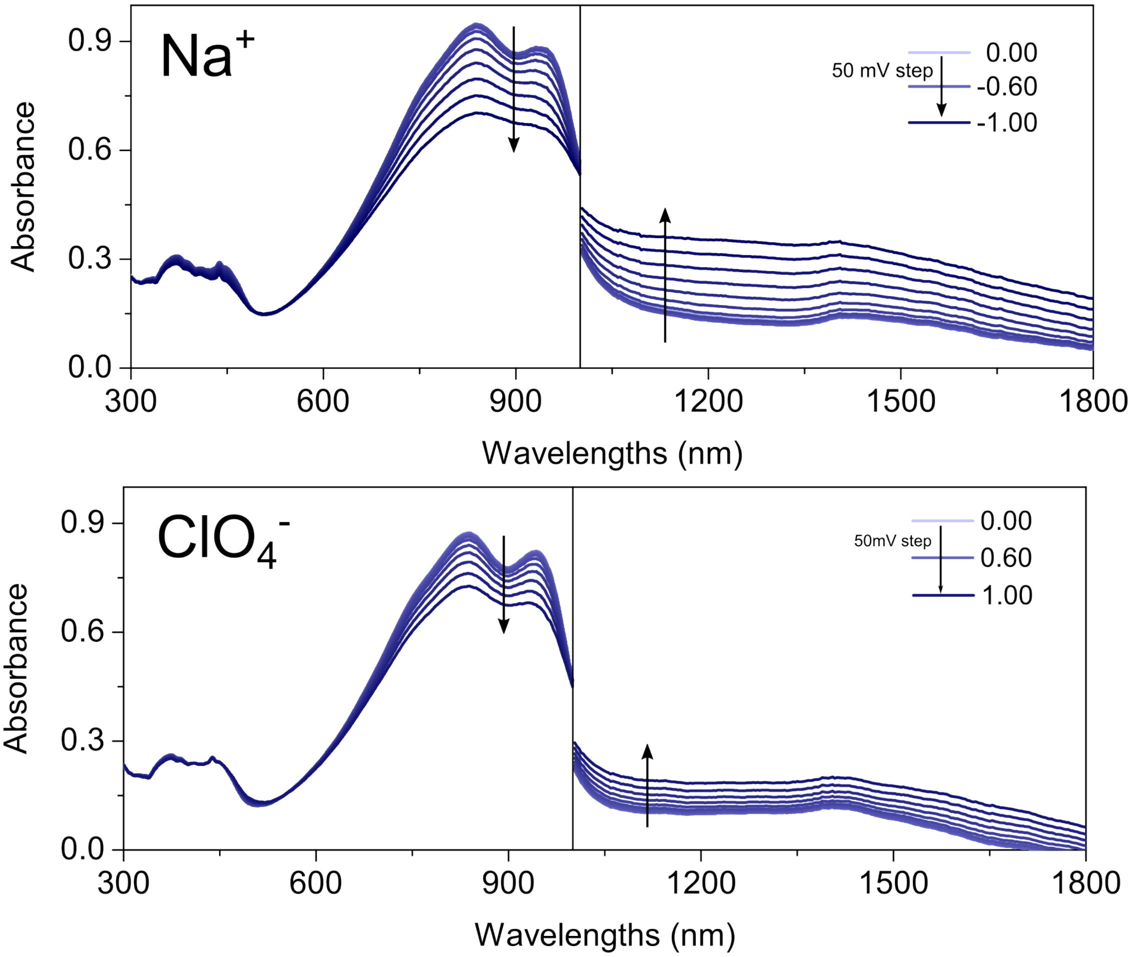

Supplementary Figure 16. Evolution of UV-Vis-NIR absorbance spectra of 2DPP-OD-TEG upon n-type and p-type electrochemical doping in aqueous electrolytes as a function of applied redox potential from 0 to -1 V and 0 to 1 V, respectively.

10. Relation between ion transfer free energy and Gutmann DN/AN

The net n-type and p-type electrochemical doping reaction is represented by equations 1 and 2 respectively

$$P + A^+_{sol} + e^- \rightarrow P^- + A^+_{Poly} \quad (1)$$

$$P + B^-_{sol} \rightarrow P^+ + B^-_{Poly} + e^- \quad (2)$$

Electrochemical doping process can be thought of as composed of two processes namely 1) ion insertion from the electrolyte into the polymer and, 2) polymer redox. The ion insertion process is analogous to an ion transfer process between two solvents and involves a free energy of transfer. The free energy of transfer of a cation, A^+ for example is difference in the solvation energies of cation in the two media i.e. polymer (Poly) and solvent (sol) written as:

$$\Delta G^{transfer}_{A^+,sol\rightarrow Poly} = \Delta G^{solv}_{A^+,Poly} - \Delta G^{solv}_{A^+,sol} \quad (3)$$

The solvation energies are strongly influenced by the coordinating properties of the solvent which are quantified by the Gutmann donor number (DN) and acceptor number (AN).

To understand the influence of DN/AN of polymer and the solvent on the ion insertion/transfer free energies, we first consider the thermodynamic cycle for the general solvation process of a cation. The free energy of solvation of ion A^+, is given by equation 4 as the difference between the free energy of the ion in the solution phase, $G_{A^+,sol}$ and gas phase, $G_{A^+,gas}$. By including the free energy of the neutral form of A, G_A and rearranging the equation, $\Delta G^{solv}_{A^+,sol}$can be expressed in terms of the free energy change of reduction reaction of A^+/A redox couple in the particular solvent, $\Delta G^{redox}_{A^+/A,sol}$.[6]

$$\Delta G^{solv}_{A^+,sol} = G_{A^+,sol} - G_{A^+,gas} = -\left(G_A - G_{A^+,sol}\right) + G_A - G_{A^+,gas} \quad (4)$$
$$= -\Delta G^{redox}_{A^+/A,sol} + G_A - G_{A^+,gas}$$

The redox potential of alkali metal cations measured in different solvents are strongly correlated with the solvent donor number (DN).[7,8] Gritzner showed that for alkali metal cations, the half wave redox potentials vary linearly with DN [9] i.e. $E_{1/2}(A^+/A) = a'_{A^+}DN + c'_{A^+}$ so that we can relate $\Delta G^{redox}_{A^+/A,sol}$ with the solvent DN using equation 5 where the coefficients a_{A^+} and c_{A^+} are related to a'_{A^+} and c'_{A^+} through the Faraday constant, F and the reference electrode potential; particularly, $a_{A^+} = Fa'_{A^+}$.

$$\Delta G^{redox}_{A^+/A,sol} = a_{A^+}DN + c_{A^+} \quad (5)$$

The solvation energies of the ion can therefore be rewritten in terms of the solvent and polymer DN as equation 6 and 7.

$$\Delta G^{solv}_{A^+,sol} = a_{A^+}.DN_{sol} + c_{A^+} + G_A - G_{A^+,gas} \quad (6)$$

$$\Delta G^{solv}_{A^+,Poly} = a_{A^+}.DN_{Poly} + c_{A^+} + G_A - G_{A^+,gas} \quad (7)$$

Using equation 3, the free energy of transfer of the ion A^+ from the solvent to the polymer is then proportional to the difference in their donor numbers. We can write down the free energy of transfer as:

$$\Delta G^{transfer}_{A^+,sol\rightarrow Poly} = a_{A^+}.(DN_{Poly} - DN_{sol}) \quad (8)$$

Gritzner calculated the values of a'_{A^+} and c'_{A^+} for various cations and the values are tabulated below[9]

Cation	a'_{A^+}	c'_{A^+}	a_{A^+}
Li^+	-0.0301	-0.836	-2904
Na^+	-0.0162	-0.903	-1563
K^+	-0.00993	-1.080	-958
Cs^+	-0.00696	-1.124	-672

Supplementary Table 2. Coefficients relating $E_{1/2}(A^+/A)$ and solvent DN for selected alkali metal cations according to ref [9]

a_{A^+} is negative which implies that when $DN_{Poly} < DN_{sol}$ and $\Delta G^{transfer}_{A^+,sol\rightarrow Poly} > 0$ leading to ion insertion process incurring an energy penalty. The larger the difference in DN, the larger the thermodynamic energy penalty and the more difficult it is to insert the ion. Further, a_{A^+} decreases with increasing cation size implying ion insertion energetics are more sensitive to the difference in DN for smaller cations.

Similarly, since anion coordination depends on Lewis acidity/electron density accepting properties of the solvent, the free energy of solvation[10] and therefore the free energy of transfer of anions between two solvents would depend on the difference in AN of the two solvents implying $\Delta G^{transfer}_{B^-,sol\rightarrow Poly}$ for anions can be written as:

$$\Delta G^{transfer}_{B^-,sol\rightarrow Poly} = b_{B^-}.(AN_{Poly} - AN_{sol}) \quad (9)$$

Here b_{B^-} is an ion dependent parameter which represents the slope of the $\Delta G^{redox}_{B/B^-,sol}$ vs. AN_{sol} graph.

11. Ion size dependent transfer characteristics of 2DPP-OD-HEX polymer

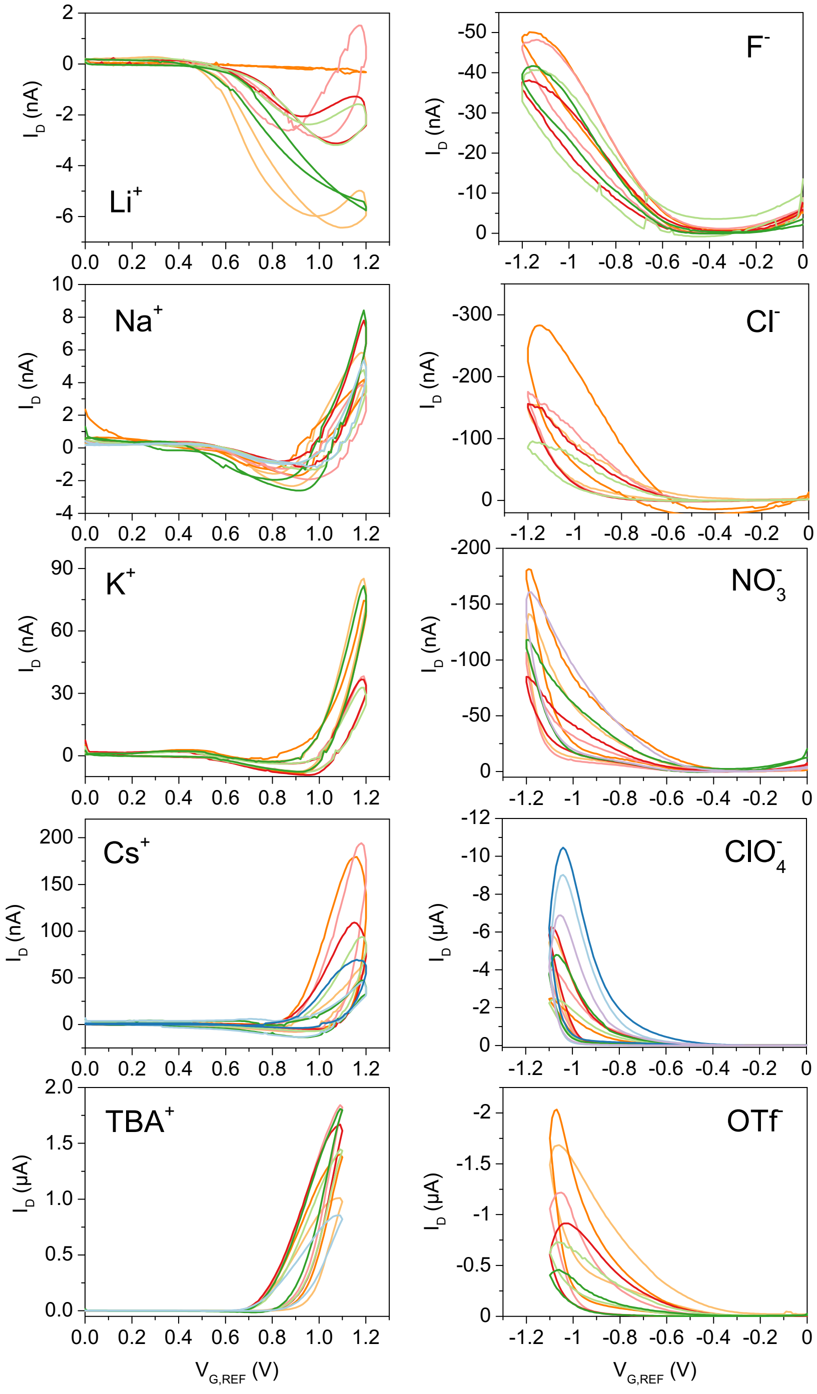

Supplementary Figure 17. N- and p-type transfer characteristics for 2DPP-OD-HEX OECTs in electrolyte solutions containing different cations and anions. The different colors represent measurements done on different devices.

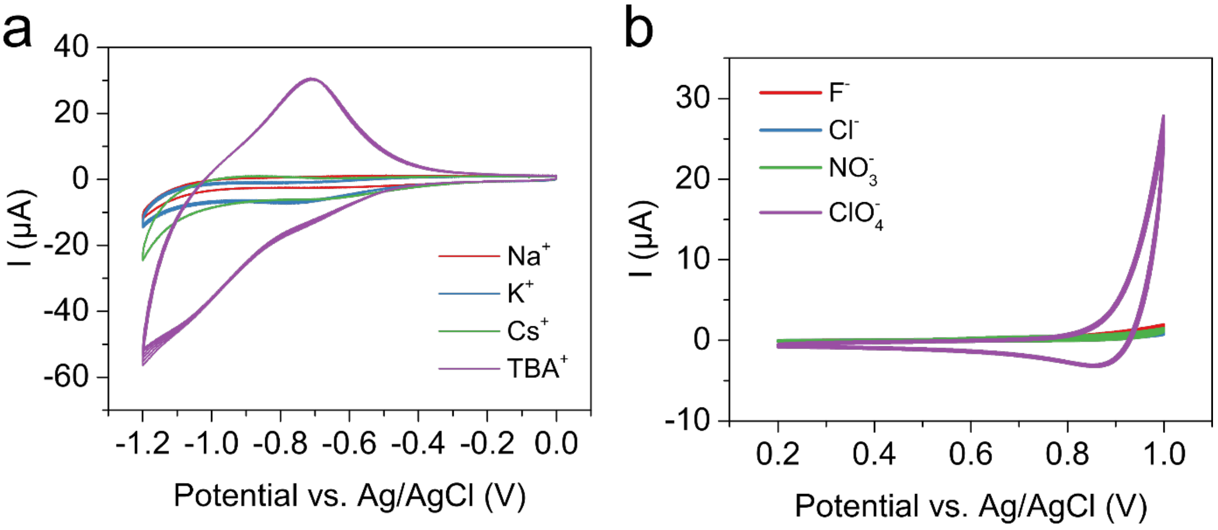

Supplementary Figure 18. CVs (5 cycles at 100mV/s) of 2DPP-OD-HEX films obtained in electrolyte solutions containing different cations and anions.

12. Passive swelling of 2DPP-OD-TEG in aqueous electrolyte

Passive swelling of 2DPP-OD-TEG was assessed using atomic force microscopy (AFM). The polymer film were prepared for swelling studies by spin coating on ITO/glass. A thin scratch was made on the film using the point of a hypodermic needle. The film was first imaged across the scratch, in the dry state. A sample AFM image of the dry film is shown in Supplementary Fig. 19a. Next, the film was covered with aqueous 0.1 M NaCl solution and allowed to soak for 1 hour. Then AFM measurements were performed on the film (Supplementary Fig. 19b) while still in contact with the electrolyte using in-liquid imaging mode, to prevent any drying/shrinking of the film. Multiple measurements were performed and mean terrace heights were calculated using Gwyddion AFM analysis tool (Supplementary Fig. 19c).[11]

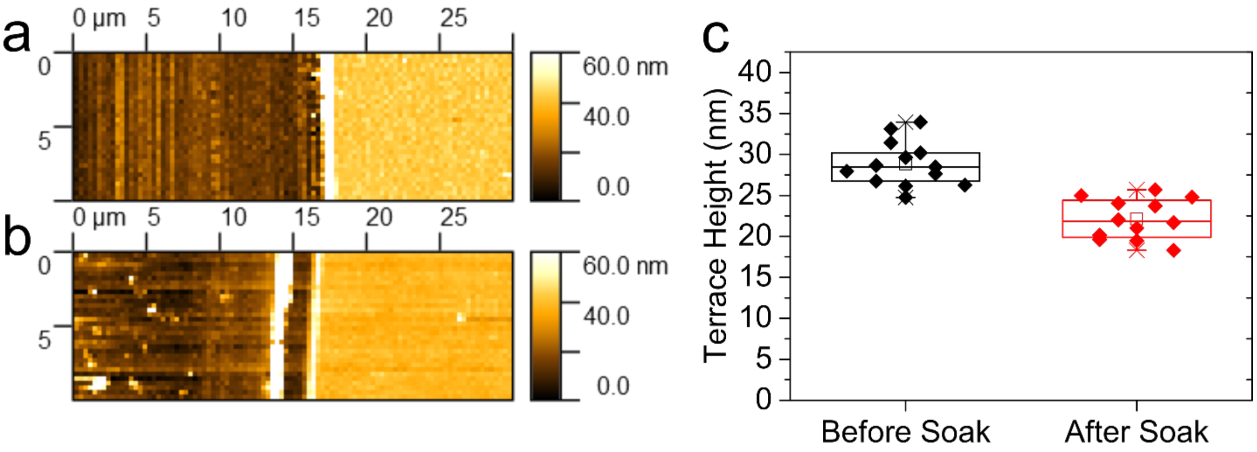

Supplementary Figure 19. (a) Sample AFM image across a scratch made on the dry polymer film (b) Sample AFM image of the same film under 0.1 M aqueous NaCl solution after having soaked in the electrolyte for 1 hour1. (c) Box plots (center line, median; box limits, 25th/75th percentiles; whiskers, outlier within 25th/75th percentile + 1.5x interquartile range; □, mean; ×, maximum and minimum values) showing the step heights calculated for multiple AFM scans of the dry film and the film under 0.1 M NaCl solution. Mean step heights of dry film and film measured under 0.1 M NaCl are 28.8±2.8 nm and 22.2±2.5 respectively

13. Correlation between n-type OECT operation and glycol sidechain weight fraction

Polymer	LUMO (eV)	glycol sidechain wt %	Cation insertion observed	n-type OECT operation	Ref.
p(gPyDPP-MeOT2)	-3.6	55.88	Yes	No	[3]
p(gPyDPP-T2)	-3.8	55.36	Yes	No	[3]
p(gDPP-TT)	-3.89	40.4	No	No	[12]
p(gDPP-T2)	-3.81	39	No	No	[12]
p(gDPP-MeOT2)	-3.6	36.12	No	No	[12]
P(bgDPP-T)	-3.69	66.9	No	No	[13]
P(bgDPP-T2)	-3.74	62.4	No	No	[13]
P(lgDPP-MeOT2)	-3.75	59.4	No	No	[13]
P(bgDPP-MeOT2)	-3.72	55.4	No	No	[13]
2DPP-OD-TEG	-3.98	20.8	Yes	Yes	this work

Supplementary Table 3. Comparison of chemical structures and OECT operation of recently reported DPP-based OECT materials

14. Donor and acceptor numbers of selected solvent molecules organized according to functional group

Functional group	Solvent	DN ($kcal.mol^{-1}$)	AN	Ref.
Alkane	Hexane	0	0	[14]
	Heptane	0	0	[14]
Amine	Ethyl amine	55	4.8	[15]
	Diethylamine	50	9.4	[14, 16]
	Triethylamine	61	1.4	[16]
	Butylamine	42	10	[14]
Nitrile	Acetonitrile	14.1	18.9	[17, 8]

	Propionitrile	16.1	19.7	[16]
	Butyronitrile	16.6	19.12	[17, 14]
Alcohol	Methanol	30	41.5	[14, 8]
	Ethanol	32	37.1	[14, 18]
	1-propanol	30	33.7	[14, 18]
	1-Butanol	29	32.2	[14, 16]
	1-pentanol	25	31	[14, 16]
	1-octanol	32	30.4	[14, 16]
	1-decanol	31	29.7	[16]
Ether	Diethyl ether	19.2	3.9	[16]
	Ethyl propanoate	17.1	6.7	[16]
	Di-n-butyl ether	19	1.55	[14]
	Glyme (1,2-Dimethoxyethane)	18.6	10.9	[19]
	Diglyme (Diethylene glycol dimethyl ether)	19.2	9.9	[19]
	Triglyme	14	10.5	[19]
	Tetraglyme	16.6	11.8	[19]
Nitro	Nitromethane	2.7	20.5	[17, 8]
	Nitroethane	5	15.8	[16, 15]
Sulfinyl	Dimethyl sulfoxide	29.8	19.3	[17, 8]
Carbonate	Dimethyl carbonate	15.17	12.28	[17, 14]
	Diethyl carbonate	15.9	8.95	[17, 14]
Ketone	Acetone	17.03	12.5	[17, 8]
	Pentan-3-one	12.6	10.3	[15]
Ester	Methyl acetate	16.3	10.7	[18]
	Ethyl acetate	17.1	9.3	[16]
	Ethyl propanoate	17.1	6.7	[16]
Carboxylic acid	Formic acid	19	83.6	[14, 18]
	Acetic acid	20	52.9	[14, 18]

Urea	1,1,3,3-Tetramethylurea	29.6	9.2	[17, 14]
Amide	Formamide	24	39.8	[8]
	N-methyl formamide	27	32.1	[8]
	N,N-Dimethylformamide	26.6	16	[17, 8]
	N,N-Diethylformamide	30.9	17.8	[17, 14]
	N,N-Dimethylacetamide	27.8	13.6	[17, 8]
	N,N-Diethylacetamide	32.2	13.6	[17, 14]
Phosphor-amide	Hexamethylphosphoramide	38.8	10.6	[8]
Phosphate	Trimethyl Phosphate	23	16.3	[18, 14]
	Triethyl Phosphate	26	17.6	[14]
	Tributyl phosphate	23.7	9.9	[14]

Supplementary Table 4. Gutmann Donor and Acceptor numbers of selected solvent molecules sorted according to their functional groups.

References

1. Cardona, C. M., Li, W., Kaifer, A. E., Stockdale, D. & Bazan, G. C. Electrochemical Considerations for Determining Absolute Frontier Orbital Energy Levels of Conjugated Polymers for Solar Cell Applications. *Adv. Mater.* **23**, 2367–2371 (2011).
2. de Leeuw, D. M., Simenon, M. M. J., Brown, A. R. & Einerhand, R. E. F. Stability of n-type doped conducting polymers and consequences for polymeric microelectronic devices. *Synth. Met.* **87**, 53–59 (1997).
3. Giovannitti, A. *et al.* Energetic Control of Redox-Active Polymers toward Safe Organic Bioelectronic Materials. *Adv. Mater.* **32**, 1908047 (2020).
4. Marcus, Y. *Ions in Solution and their Solvation*. (Wiley, Hoboken, 2015).
5. Pearson, R. G. Absolute electronegativity and hardness: application to inorganic chemistry. *Inorg. Chem.* **27**, 734–740 (1988).

6. Aetukuri, N. B. *et al.* Solvating additives drive solution-mediated electrochemistry and enhance toroid growth in non-aqueous Li–O2 batteries. *Nat. Chem.* **7**, 50–56 (2015).
7. Gritzner, G. Solvent effects on redox potentials: Studies in N-methylformamide. *J. Electroanal. Chem. Interfacial Electrochem.* **144**, 259–277 (1983).
8. Fawcett, W. R. Acidity and basicity scales for polar solvents. *J. Phys. Chem.* **97**, 9540–9546 (1993).
9. Gritzner, G. Solvent effects on half-wave potentials. *J. Phys. Chem.* **90**, 5478–5485 (1986).
10. Parker, A. J., Mayer, U., Schmid, R. & Gutmann, V. Correlation of solvent effects on rates of solvolysis and SN2 reactions. *J. Org. Chem.* **43**, 1843–1854 (1978).
11. Nečas, D. & Klapetek, P. Gwyddion: an open-source software for SPM data analysis. *Open Phys.* **10**, 181–188 (2012).
12. Moser, M. *et al.* Polaron Delocalization in Donor–Acceptor Polymers and its Impact on Organic Electrochemical Transistor Performance. *Angew. Chem. Int. Ed.* **60**, 7777–7785 (2021).
13. Jia, H. *et al.* Engineering donor–acceptor conjugated polymers for high-performance and fast-response organic electrochemical transistors. *J. Mater. Chem. C* **9**, 4927–4934 (2021).
14. Marcus, Y. The properties of organic liquids that are relevant to their use as solvating solvents. *Chem. Soc. Rev.* **22**, 409 (1993).
15. Schmid, R. Re-interpretation of the solvent dielectric constant in coordination chemical terms. *J. Solut. Chem.* **12**, 135–152 (1983).
16. Marcus, Y. *The properties of solvents*. (Wiley, Chichester, 1998).
17. Laurence, C. & Gal, J.-F. *Lewis Basicity and Affinity Scales : Data and Measurement*. (John Wiley & Sons Ltd, 2010).
18. Reichardt, C. *Solvents and Solvent Effects in Organic Chemistry*. (Wiley-VCH, Weinheim, 2003).

19. Brouillette, D., Perron, G. & Desnoyers, J. E. Apparent Molar Volume, Heat Capacity, and Conductance of Lithium Bis(trifluoromethylsulfone)imide in Glymes and Other Aprotic Solvents. *J. Solut. Chem.* **27**, 151–182 (1998).
